# Supplementary material for: Identification and functional analysis of non-coding regulatory small RNA FenSr3 in Bacillus amyloliquefaciens LPB-18
Source: PeerJ. 2023 May 15;11:e15236. doi: 10.7717/peerj.15236 (PMC10194069; doi:10.7717/peerj.15236)
Supplement: Supplemental Information 4 [file peerj-11-15236-s004.zip › KO/CK-vs-T1_map/map04622.html]

KEGG PATHWAY: RIG-I-like receptor signaling pathway - Reference pathway


|  |  |
| --- | --- |
| **RIG-I-like receptor signaling pathway - Reference pathway** |  |

[
Pathway menu
| Organism menu
| Pathway entry
| Show description
| User data mapping
]

Specific families of pattern recognition receptors are responsible for detecting viral pathogens and generating innate immune responses. Non-self RNA appearing in a cell as a result of intracellular viral replication is recognized by a family of cytosolic RNA helicases termed RIG-I-like receptors (RLRs). The RLR proteins include RIG-I, MDA5, and LGP2 and are expressed in both immune and nonimmune cells. Upon recognition of viral nucleic acids, RLRs recruit specific intracellular adaptor proteins to initiate signaling pathways that lead to the synthesis of type I interferon and other inflammatory cytokines, which are important for eliminating viruses.

|  |  |  |
| --- | --- | --- |
| Reference pathway | 184% 150% 122% 100% 82% 67% 55% | 图片下载 |
